# Regulation of the opposing (p)ppGpp synthetase and hydrolase activities in a bifunctional RelA/SpoT homologue from *Staphylococcus aureus*

**DOI:** 10.1371/journal.pgen.1007514

**Published:** 2018-07-09

**Authors:** Fabio Lino Gratani, Petra Horvatek, Tobias Geiger, Marina Borisova, Christoph Mayer, Iwan Grin, Samuel Wagner, Wieland Steinchen, Gert Bange, Ana Velic, Boris Maček, Christiane Wolz

**Affiliations:** 1 Interfaculty Institute of Microbiology and Infection Medicine, University of Tuebingen, Tuebingen, Germany; 2 German Centre for Infection Research, Partner Site Tuebingen, Tuebingen, Germany; 3 Center for Synthetic Microbiology (SYNMIKRO) & Dept. of Chemistry, Philipps-University, Marburg, Germany; 4 Quantitative Proteomics and Proteome Center Tuebingen, Tuebingen, Interfaculty Institute for Cell Biology, University of Tuebingen, Tuebingen, Germany; The University of Texas Health Science Center at Houston, UNITED STATES

## Abstract

The stringent response is characterized by (p)ppGpp synthesis resulting in repression of translation and reprogramming of the transcriptome. In *Staphylococcus aureus*, (p)ppGpp is synthesized by the long RSH (RelA/SpoT homolog) enzyme, Rel_*Sau*_ or by one of the two short synthetases (RelP, RelQ). RSH enzymes are characterized by an N-terminal enzymatic domain bearing distinct motifs for (p)ppGpp synthetase or hydrolase activity and a C-terminal regulatory domain (CTD) containing conserved motifs (TGS, DC and ACT). The intramolecular switch between synthetase and hydrolase activity of Rel_*Sau*_ is crucial for the adaption of *S*. *aureus* to stress (stringent) or non-stress (relaxed) conditions. We elucidated the role of the CTD in the enzymatic activities of Rel_*Sau*_. Growth pattern, transcriptional analyses and *in vitro* assays yielded the following results: i) *in vivo*, under relaxed conditions, as well as *in vitro*, the CTD inhibits synthetase activity but is not required for hydrolase activity; ii) under stringent conditions, the CTD is essential for (p)ppGpp synthesis; iii) Rel_*Sau*_ lacking the CTD exhibits net hydrolase activity when expressed in *S*. *aureus* but net (p)ppGpp synthetase activity when expressed in *E*. *coli*; iv) the TGS and DC motifs within the CTD are required for correct stringent response, whereas the ACT motif is dispensable, v) Co-immunoprecipitation indicated that the CTD interacts with the ribosome, which is largely dependent on the TGS motif. In conclusion, Rel_*Sau*_ primarily exists in a synthetase-OFF/hydrolase-ON state, the TGS motif within the CTD is required to activate (p)ppGpp synthesis under stringent conditions.

## Author summary

The stringent response is a general stress response, which allows bacteria to survive nutrient limited conditions and to better tolerate antibiotic treatment. In the human pathogen, *Staphylococcus aureus*, the stringent response plays an important role for virulence, phagosomal escape and antibiotic tolerance. The response is initiated by the synthesis of the nucleotide derivative (p)ppGpp which in turn leads to growth arrest and reprogramming of gene expression. However, a rapid and controlled inactivation of these growth inhibitory molecules is equally important for the organism. (p)ppGpp synthesis as well as hydrolysis is accomplished by a bi-functional RelA/SpoT homolog, Rel_*Sau*_ bearing distinct synthetase, hydrolase and sensory domains. We elucidated how the C-terminal sensory domain of Rel_Sau_ controls the intermolecular switch between hydrolase and synthetase activities in *S*. *aureus*. The switch is crucial for the appropriate response of *S*. *aureus* to adapt to changing environment encountered during infection.

## Introduction

Bacteria react to nutrient limitation via a stress response that is characterized by the synthesis of pyrophosphorylated GTP (pppGpp) or GDP (ppGpp) (previously reviewed in [1,2,3,4,5,6,7,8,9,10,11]). Synthesis of (p)ppGpp, induced under these stress conditions (stringent conditions), results in many physiological changes, including inhibition of rRNA synthesis, replication and translation but also activation or repression of various genes. In many pathogenic bacteria, (p)ppGpp influences virulence, persistence and host interaction (see reviews [9,10]).

(p)ppGpp is synthesized by cytoplasmic enzymes that contain a conserved synthetase domain. RelA of *Escherichia coli* was the first such enzyme described and has been shown to synthesize (p)ppGpp under conditions of amino acid limitation [12]. *E*. *coli* and many other gram-negative bacteria possess an additional enzyme, SpoT, that possesses (p)ppGpp synthetase and hydrolase activities. The (p)ppGpp synthetase activity of SpoT is stimulated by various conditions, e.g. fatty acid deprivation [13,14]. In Firmicutes, homologous enzymes (Rel) constitute a distinct class of (p)ppGpp synthetases [11,15,16]. RelA, SpoT and Rel enzymes all belong to RSH (for RelA/SpoT homolog) superfamily [15,17]. Similar to SpoT, the Rel enzymes from Firmicutes are bifunctional proteins with (p)ppGpp synthetase and hydrolase activities; however, similar to RelA, the synthetase activity of these enzymes is stimulated upon amino acid starvation [18,19]. RSH enzymes share a multi-domain architecture with a C-terminal regulatory domain (CTD) and an N-terminal enzymatic domain (NTD) containing synthetase and hydrolase motifs. The only available crystal structure of an RSH enzyme is that of the NTD of Rel from *Streptococcus equisimilis* [20]. The structure indicates two conformations of the enzyme, corresponding to the reciprocal active states of the enzyme: (p)ppGpp-synthetase-ON/hydrolase-OFF (stringent) and synthetase-OFF/hydrolase-ON (relaxed). It has been proposed that the CTD is involved in reciprocal regulation of the enzymatic states. The current model suggests that under non-stringent (relaxed) conditions, the interaction of the CTD with the NTD maintains the enzyme in the synthetase-OFF/hydrolase-ON conformation [21,22,23]The CTD of RelA stimulates (p)ppGpp synthesis in a ribosome-dependent manner when uncharged tRNA, as a consequence of amino acid limitation, is located in the ribosomal A-site [24,25]. Interestingly, Rel from *S*. *equisimilis* is responsive to amino acid starvation only within its native genetic background and not when expressed in *E*. *coli* [13,21]. Bioinformatic analyses have revealed the presence of three conserved motifs within the CTDs of RSHs: TGS, ACT and DC. The TGS motif (named after the presence in ThrRS, GTPases, and SpoT) was shown to be responsible for the interaction of SpoT with the acyl-carrier protein. The ACT motif (named after three of the allosterically regulated enzymes in which this domain is found: aspartate kinase, chorismate mutase and TyrA) was proposed to be a conserved regulatory ligand-binding fold [26,27]. Recently, major insights into the ribosome-RelA structure were provided by cryo-EM analyses [28,29,30]. The structures revealed that RelA adopts an open conformation in which the CTD is intertwined around an A-site tRNA within the intersubunit cavity of the ribosome, and the NTD extends into the solvent. The structures support a model in which association of monomeric RelA with the ribosome relieves the autoinhibitory effect of the CTD on the NTD. It was hypothesized that autoinhibition in the unbound state is mediated by oligomerization of RelA. Oligomerization was previously demonstrated to occur via a conserved aspartate-cysteine motif (DC) in the CTD [31,32,33]. Interaction of monomeric RelA with the ribosome and putative RelA oligomerization in the unbound state indicate that the switching of enzymatic activities occurs via a complex mechanism that has not yet been elucidated.

In the human pathogen *Staphylococcus aureus*, the stringent response plays an important role in virulence [18], phagosomal escape [34] and antibiotic tolerance [35]. In *S*. *aureus*, in addition to Rel_*Sau*_, two enzymes with (p)ppGpp synthetase activity (RelP and RelQ) are present. These enzymes form homotetramers that lack the CTD and the hydrolase domain [36,37,38] and are transcriptionally induced under conditions of cell-wall stress [35]. The basal (p)ppGpp level produced by these enzymes is controlled by the hydrolase activity of Rel_*Sau*_ [35]. The phenotypic consequences of (p)ppGpp accumulation vary among species and can be mediated by different mechanisms. In *S*. *aureus*, as in other Firmicutes, (p)ppGpp regulates transcription by an indirect mechanism that strongly relies on the lowering of intracellular GTP levels [39,40,41]. Low GTP levels lead to de-repression of the CodY regulon. CodY, when loaded with GTP and branched-chain amino acids, acts as a repressor of a variety of genes, e.g., genes involved in amino acid synthesis and virulence [41] A decrease in GTP levels could also lead to the repression of sensitive GTP-initiating promoters (e.g., those of stable RNA genes) [42,43]. All these studies illustrate the complex role of (p)ppGpp during the bacterial life cycle. The cellular concentration of (p)ppGpp has to be tightly regulated not only to support survival under stressed conditions but also to avoid toxicity under relaxed conditions. The molecular switch between the synthetase and hydrolase activities of Rel_*Sau*_ is crucial for the maintenance of this balance.

Here, we aim to elucidate the role of the CTD in controlling the activity of Rel of the major human pathogen *S*. *aureus* (Rel_*Sau*_) *in vivo*. We show that the (p)ppGpp synthetase activity is restricted in *S*. *aureus* and that the synthetase is activated only upon interaction of the CTD with ribosomal partners under stringent conditions. The TGS and DC motifs within the CTD are essential for the enzymatic switch to the synthetase-ON state and play a major role in the interaction between Rel_*Sau*_ and the translational apparatus.

## Results

### CTD-deleted Rel_*Sau*_ is in a synthetase-OFF/hydrolase-ON state in its native *S*. *aureus* background

**Fig 1:**
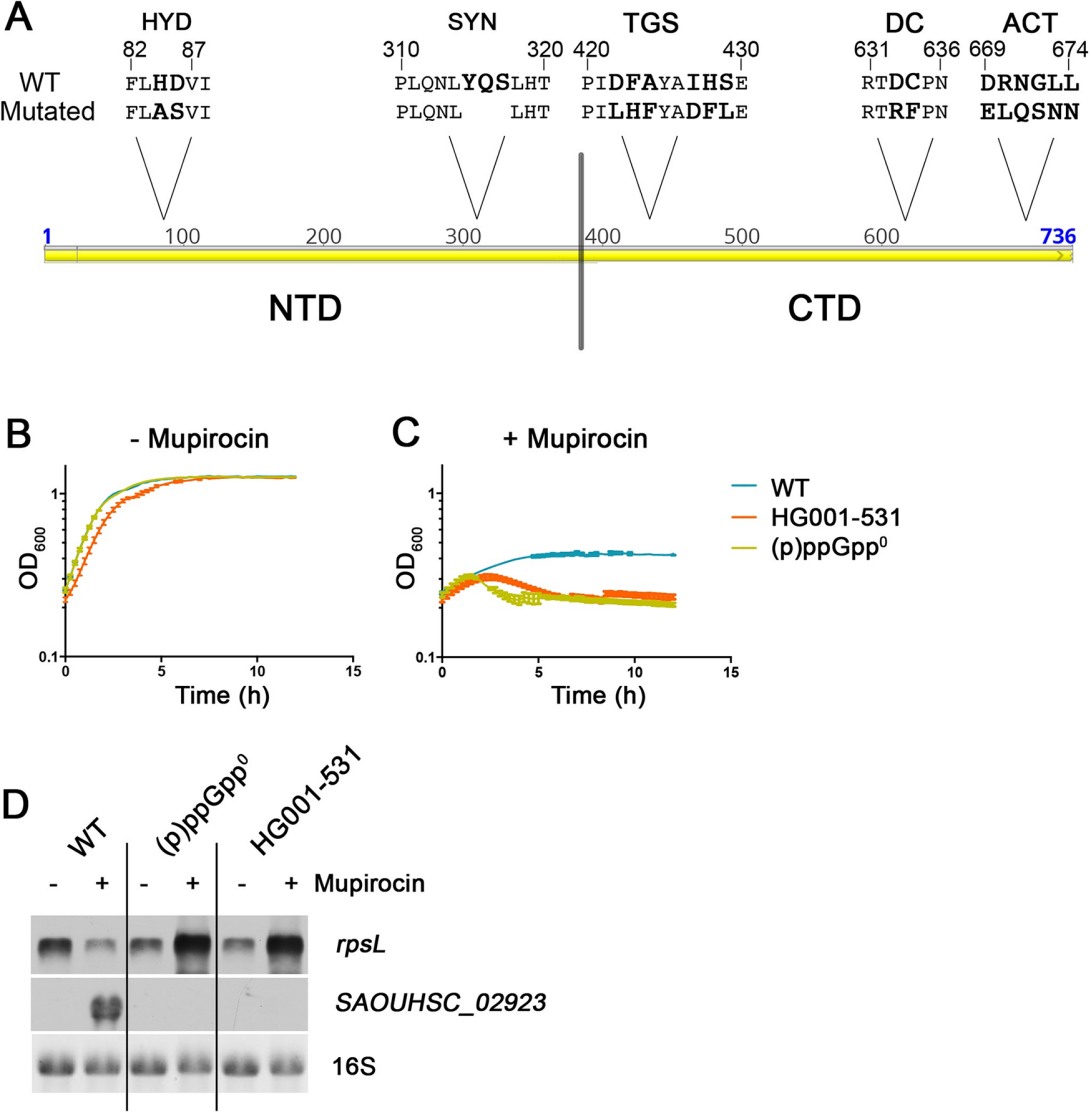
Experimental set-up for synthetase activity in *S*. *aureus*. (A) The molecular architecture of Rel_*Sau*_ including conserved and mutated motifs. *S*. *aureus* strains HG001 wild type (WT), (p)ppGpp^0^ and HG001-531 (*rel* CTD deleted) were grown in rich medium to OD_600_ = 0.3 and split in cultures with and without 0.3 μg/ml mupirocin. Growth was monitored after reaching OD_600_ = 0.3 without (B) or with mupirocin (C).For Northern analysis (D) RNA was isolated from bacteria 30 minutes after reaching OD_600_ = 0.3 and hybridized with digoxigenin-labelled probes specific for gene encoding ribosomal protein RpsL and the CodY target gene SAOUHSC_02923. The 16S rRNA detected in the ethidium bromide-stained gels is indicated as loading control in the bottom lane.

We aimed to analyze the role of the CTD of Rel_*Sau*_ in the stringent response in *S*. *aureus*. In Rel_*Sau*_ the canonical domains and motifs could be identified through alignment with RelA and SpoT from *E*. *coli* (Fig 1A). We first established a readout system for (p)ppGpp activity. To this end, we analyzed strain HG001 (wild type) as well as an isogenic mutant of this strain that carries mutations in all three (p)ppGpp enzymes (full deletion of *rel*, synthetase mutation in *relP* and *relQ*) and thus is unable to synthesize pppGpp (designated (p)ppGpp^0^) [35]. The mutant exhibits no phenotypic difference compared to the wild type when grown in rich medium (Fig 1B). The stringent response can be evoked by mupirocin, an inhibitor of isoleucyl-tRNA synthetase [18,34]. (p)ppGpp synthesis results in higher tolerance towards mupirocin. The (p)ppGpp^0^ strain exhibited a typical decline in OD_600_ when treated with mupirocin (Fig 1C). Furthermore, synthesis of (p)ppGpp results in repression of genes coding for ribosomal proteins (e.g., *rpsL*) and de-repression of the CodY target genes (e.g., SAOUHSC_02923, a putative amino acid transporter) (Fig 1D). Therefore, we used the enhanced mupirocin tolerance and typical transcription pattern (*rpsL* down, SAOUHSC_02923 up) as a readout for (p)ppGpp synthesis in *S*. *aureus*. As a first approach, we deleted the CTD of the wild-type Rel_*Sau*_ (SAOUHSC_01742) to generate strain HG001-531. In contrast to a full-length *rel* deletion (Geiger et al., 2014a), truncation of the CTD had only a slight effect on growth (Fig 1C). It has been previously shown that Rel_*Sau*_ is essential due to its hydrolase function [35]. Thus, the CTD does not seem to impede the hydrolase activity *in vivo*. Northern blot analysis revealed that the CTD mutant was unable to elicit a mupirocin-induced stringent response (Fig 1B). The transcriptional pattern of the marker genes *rpsL* and SAOUHSC_02923 as well as the mupirocin tolerance (Fig 1D) of the CTD mutant were indistinguishable from those of the (p)ppGpp^0^ strain. This finding is consistent with the general assumption that the CTD is required for sensing amino acid deprivation. However, hydrolase activity seems to be hardly effected by the CTD.

**Fig 2:**
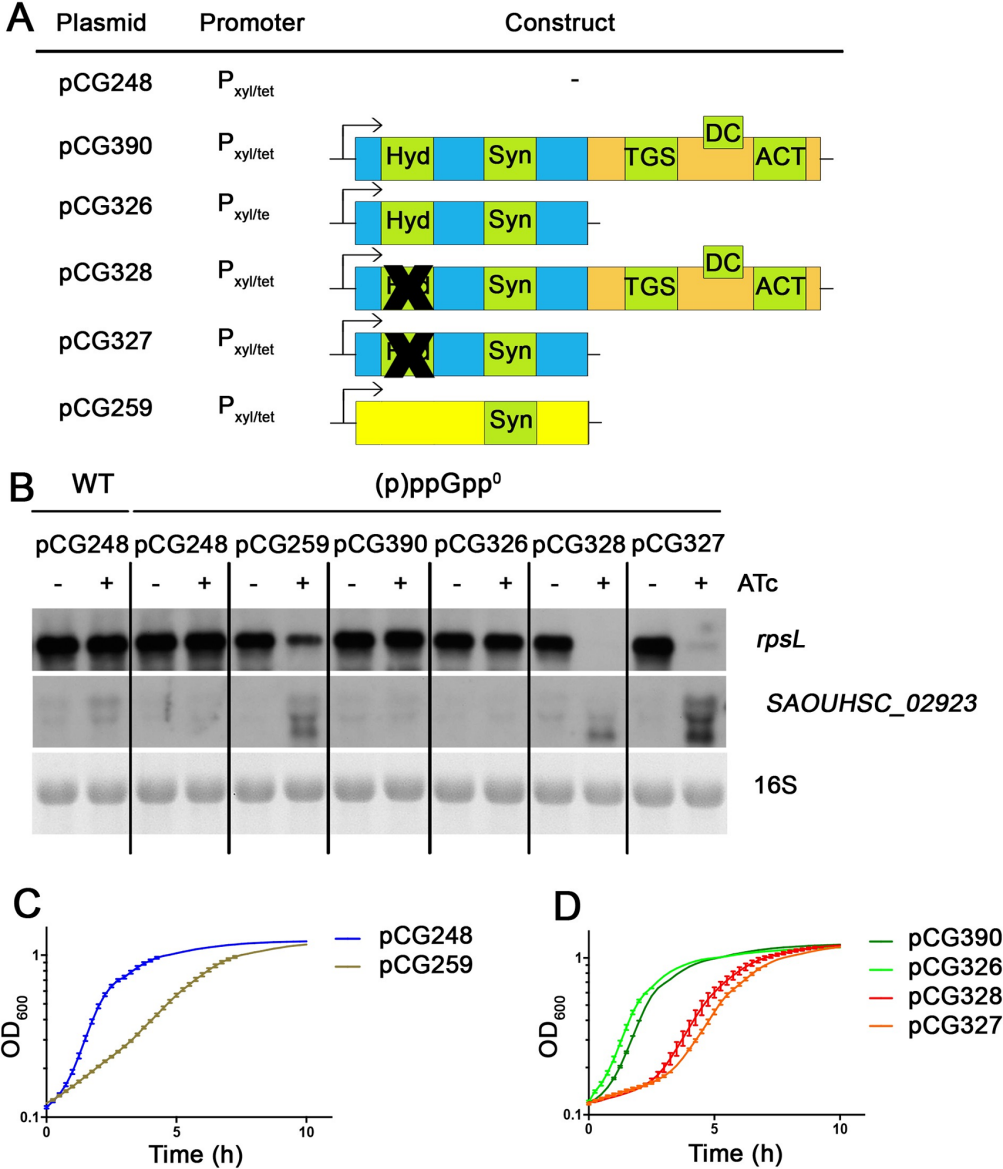
Influence of the CTD on the synthetase activity *in vivo*. ATc inducible *rel* with and without CTD or hydrolase domain and *relQ* (A) were expressed in the (p)ppGpp^0^ mutant and compared to WT HG001. The black crosses (A) represent mutations as indicated in Fig 1A. Strains were grown in rich medium to OD_600_ = 0.3 and then split in cultures with and without 0.1 μg/ml ATc. For Northern analysis (B) RNA was isolated from bacteria 30 minutes after reaching OD_600_ = 0.3 and hybridized with digoxigenin-labelled probes. The 16S rRNA detected in the ethidium bromide-stained gels is indicated as loading control in the bottom lane. *rpsL* and SAOUHSC_02923 are control markers for the induction by (p)ppGpp Growth was monitored after addition of 0.1 μg/ml ATc. The (p)ppGpp^0^ was complemented with different plasmids: empty vector (pCG248) and *relQ* (pCG259), as controls (C), and different *rel* constructs (D).

Next, we complemented the (p)ppGpp^0^ strain with anhydrotetracycline (ATc)-inducible full-length and truncated *rel* constructs (Fig 2A) and analyzed the effects under relaxed growth conditions (exponential growth phase in nutrient-rich medium). As a positive control, we induced *relQ* expression. RelQ is a small synthetase without a regulatory CTD and thus can activate the stringent response in a (p)ppGpp^0^ mutant by transcriptional induction alone. This activation was demonstrated by downregulation of *rpsL* and upregulation of SAOUHSC_02923 (Fig 2B) and by the immediate growth arrest after *relQ* induction (Fig 2C).

In contrast to *relQ*, transcriptional induction of full-length *rel* showed no effect on the transcription of marker genes or on growth (Fig 2B and 2D). This finding confirms that additional post-transcriptional activation is required to activate the synthetase activity. Induction of a construct lacking the CTD also failed to induce the stringent response phenotype (Fig 2B and 2D). At first glance, these results may indicate that under relaxed conditions, the enzymatic domain of Rel_*Sau*_, with or without the CTD, is tightly held in a synthetase-OFF conformation. Alternatively, the hydrolase might be hyperactive, so any (p)ppGpp synthesized would be immediately degraded. To test this hypothesis, we mutated the hydrolase domain in full-length and CTD-deleted *rel* constructs. Indeed, both full-length and truncated *rel* lacking the hydrolase domain elicited a stringent response pattern similar to that of the wild type, as indicated by transcriptional and growth analyses (Fig 2B and 2D). Thus, we presume that there might be some synthetase activity under relaxed growth conditions. However, due to hydrolase activity, any (p)ppGpp present is efficiently degraded under these conditions.

### CTD of Rel_*Sau*_ does not impact hydrolase activity *in vivo*

**Fig 3:**
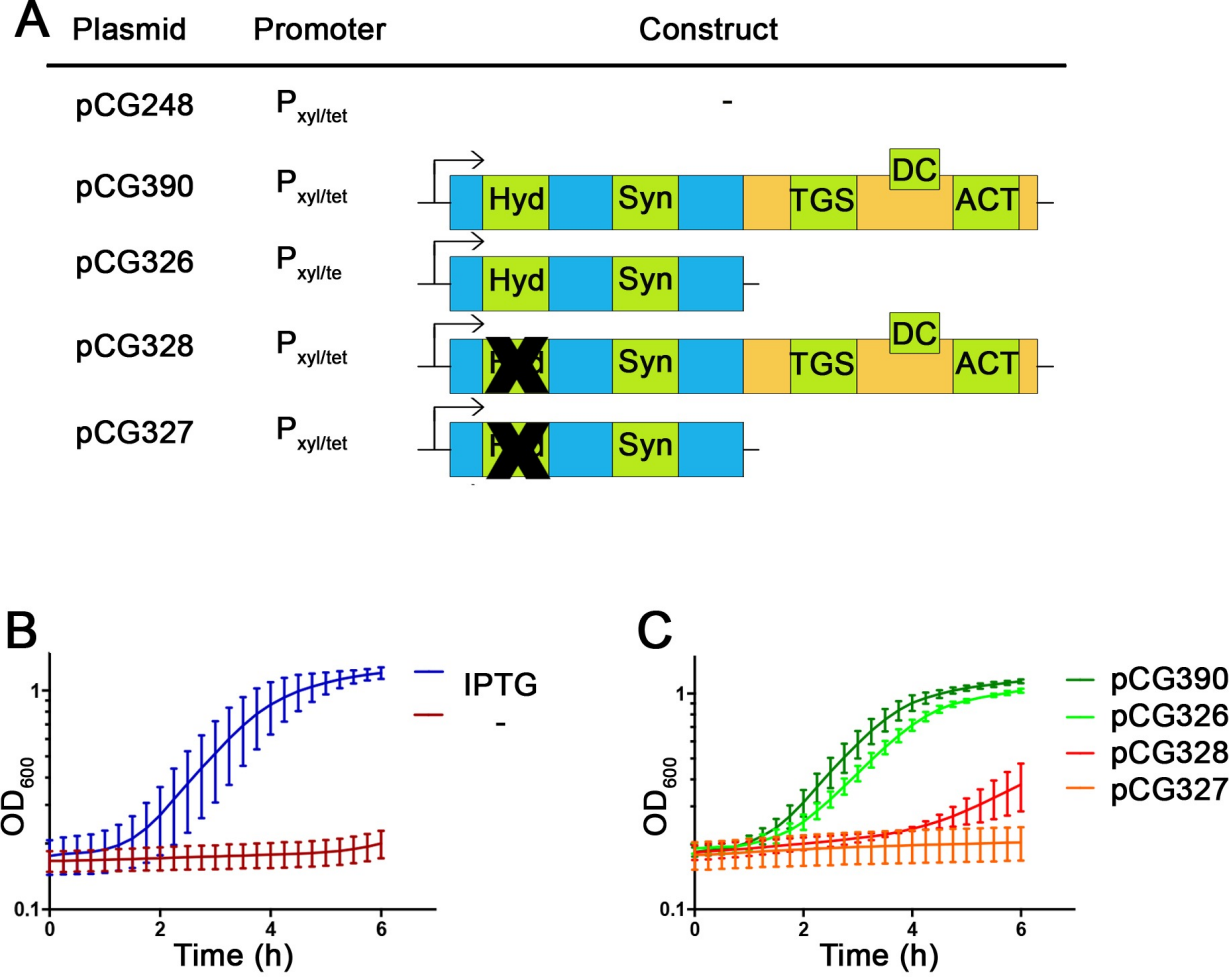
CTD of Rel_*Sau*_ has no impact on hydrolase activity *in vivo*. The conditional *rel* mutant HG001-55 (inducible by IPTG) was complemented with ATc inducible *rel* constructs (A). Strains were grown during preculture in presence of IPTG (0.5 mM) and were diluted to an initial OD_600_ = 0.1 and growth monitored over time. As control, strain complemented with empty vector was grown with and without IPTG to illustrate the essentially of the Rel hydrolase (B). *Rel* constructs were grown without IPTG but addition of ATc (0.1 μg/ml) (C).

To analyze the hydrolase activity of Rel_*Sau*_ *in vivo*, we used a conditional *rel* mutant strain (HG001-55) [18] in which genomic *rel* was placed under an IPTG-inducible promoter complemented with different rel constructs (Fig 3A). Without IPTG, the *rel* mutant is unable to grow (Fig 3B) because it cannot degrade the (p)ppGpp synthesized by RelP and RelQ [35]. We introduced ATc-inducible full-length or truncated *rel* into the conditional *rel* mutant and monitored growth after ATc induction. As expected, constructs with mutated hydrolase could not rescue the growth defect of HG001-55 (Fig 3C). However, full-length and CTD-truncated *rel*, with intact hydrolase, fully complemented the growth defect of the conditional *rel* mutant. These results show that the hydrolase was constitutively active, independent of the presence of the CTD. In summary, the data indicate that under relaxed conditions, the wild-type Rel_*Sau*_ enzyme, with or without sensory domain, is tightly held in the hydrolase-ON state.

### Role of the CTD in the enzymatic activity of Rel_*Sau*_ *in vitro*

**Fig 4:**
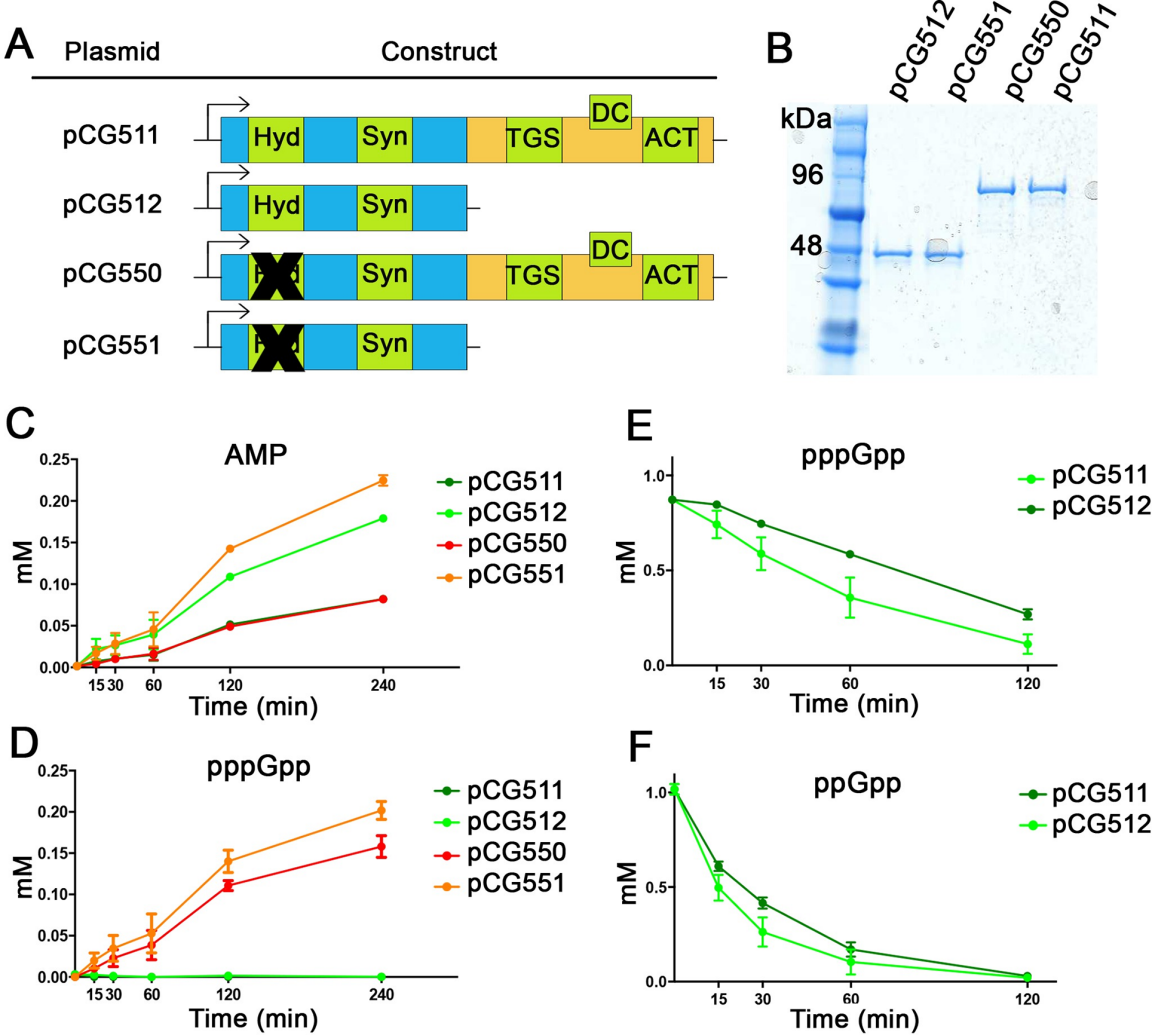
Role of the CTD on the enzymatic activity *in vitro*. 2 μM of purified proteins with and without CTD or hydrolase domain (A) were loaded on SDS-PAGE, Rel_*Sau*_ (85 KDa) and CTD-deleted (48KDa) (B) and used to assay synthetase (C, D) and hydrolase activity (E, F). The black crosses (A) represent mutations as indicated in Fig 1A. Synthetase activity was determined in the presence of the two substrates ATP and GTP and 2 μM of enzymes. The reaction products AMP (C) and pppGpp (D) were monitored over time at 37°C by HPLC-MS. Hydrolase activity was assayed in the presence of pppGpp (E) or ppGpp (F) and 0.1 μM of enzymes. Decrease of 1 mM of pppGpp or ppGpp was monitored for 120 minutes at 37°C.

To confirm the data from the *in vivo* experiments under relaxed conditions, full-length or CTD-truncated Rel_*Sau*_ proteins (with or without hydrolase domains) were purified and tested *in vitro* for enzymatic activities (Fig 4A and 4B). In the synthetase reaction, pyrophosphate is transferred from ATP to GTP, yielding AMP and pppGpp. The presence of both products was measured by HPLC-MS. AMP production was detectable with all constructs (Fig 4C); however, the AMP levels were significantly higher for the constructs that lacked the CTD (Fig 4C), indicating that the CTD negatively interferes with synthetase activity. Interestingly, pppGpp production was not detected for constructs with intact hydrolase (Fig 4D). However, proteins with mutated hydrolases synthesized detectable amounts of (p)ppGpp. The enzyme lacking the CTD showed slightly higher pppGpp synthetase activity than the full-length Rel_*Sau*_ supporting the inhibitory effect of the CTD on the synthetase domain. Thus, Rel_*Sau*_ exhibits strong hydrolase activity, which prevents pppGpp accumulation. This finding was confirmed by the rapid degradation of pppGpp (Fig 4E) and ppGpp (Fig 4F) by full-length and CTD deleted Rel_Sau_. Notably, Rel_*Sau*_ preferentially degraded ppGpp over pppGpp. The CTD apparently has a minor impact on hydrolase activity.

### Role of the CTD of *Rel*_*Sau*_ in *E*. *coli*

**Fig 5:**
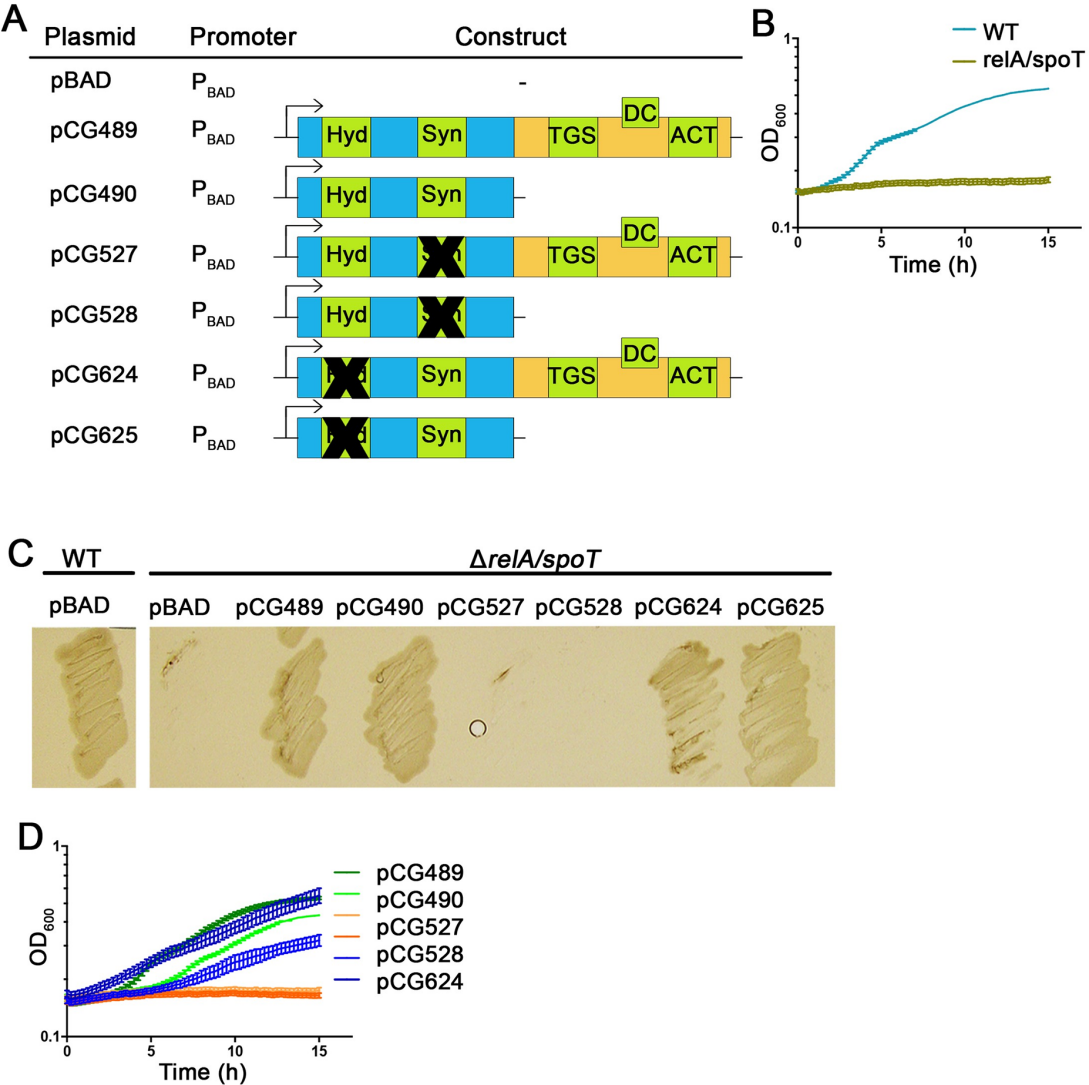
**Role of CTD of Rel_*Sau*_ in *E*. *coli*.** *rel* from *S*. *aureus* with and without CTD, hydrolase or synthetase domain (A) were expressed in *relA*/*spoT* mutant of *E*. *coli* MG1655. The black crosses (A) represent mutations as indicated in Fig 1A. As controls WT and *relA*/*spoT* mutant were transformed with empty vector (B and C). Strains were grown under stringent conditions streaked on M9 agar plates (C) or M9 medium for growth analysis (D). Under these conditions the *relA*/*spoT* mutant was unable to grow.

Our analysis of Rel_*Sau*_ in its native background seemed to be inconsistent with the results of previous studies, in which different CTD-deleted enzymes from other organisms were expressed in *E*. *coli* [20,21,44,45]. These studies indicated that RSH enzymes that lack CTDs are in a synthetase-ON/hydrolase-OFF state. Thus, based on these studies, we also expressed full-length and CTD-deleted *rel* in *E*. *coli* using an arabinose-inducible promoter. We tested the capacity of different *rel* constructs (Fig 5A) to complement the defective phenotype of MG1655, a *relA/spoT* mutant, under stringent conditions (Fig 5B and 5C). Full-length and CTD-deleted Rel_*Sau*_ were able to complement the *relA/spoT* mutation. The complementation could be attributed to (p)ppGpp synthetase activity: mutation within the synthetase domain abolished complementation, whereas mutation within the hydrolase domain did not affect the complementation assay. Thus, in *E*. *coli*, CTD-deleted Rel_*Sau*_, similar to other RSH enzymes, is predominantly in a synthetase-ON/hydrolase-OFF state, whereas in *S*. *aureus*, this enzyme is primarily in a hydrolase-ON state.

### Role of CTD motifs for sensing *in vivo*

**Fig 6:**
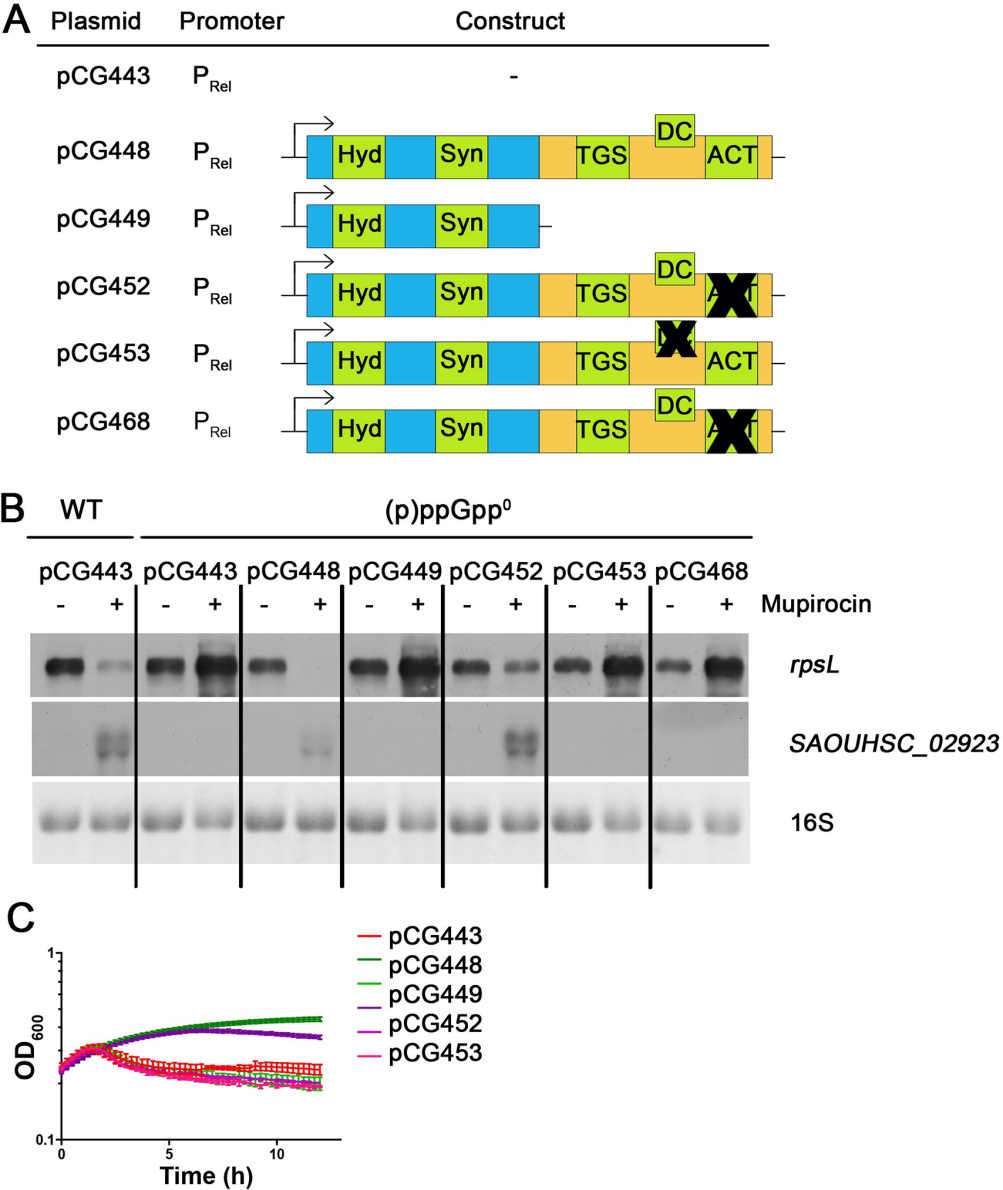
Role of CTD motifs for sensing of Rel_*Sau*_ *in vivo*. (p)ppGpp^0^ mutant was complemented with different *rel* constructs expressed from the native promoter (A). The black crosses (A) represent mutations as indicated in Fig 1A. Strains were grown in rich medium to OD_600_ = 0.3 and then split in cultures with and without 0.3 μg/ml mupirocin. For Northern analysis (B) RNA was isolated from cells 30 minutes after reaching OD_600_ = 0.3 and hybridized with digoxigenin-labeled probes. The 16S rRNA detected in the ethidium bromide-stained gels is indicated as loading control in the bottom lane. Growth was monitored after reaching OD_600_ = 0.3 after which 0.3. μg/ml mupirocin was added (C).

Within the CTDs of RSHs, several conserved motifs can be identified. The conserved TGS, DC and ACT motifs of Rel_*Sau*_ were predicted based on sequence alignments, and the critical residues of these motifs were mutated (Fig 1A). Wild-type and CTD-mutated *rel* were cloned to be under the control of the native *rel* promoter and introduced into the (p)ppGpp^0^ strain (Fig 6A). The stringent response upon mupirocin treatment was analyzed by Northern blotting and growth analysis (Fig 6B and 6C). A (p)ppGpp^0^ strain containing the empty vector showed the typical decrease in OD_600_ after mupirocin treatment. Induction of full-length *rel* in the (p)ppGpp^0^ mutant fully complemented the mutant phenotype, whereas the CTD-deleted *rel* was unable to do so. Mutation of the ACT motif resulted in slightly impaired complementation. However, mutation of the TGS or DC motif resulted in complete inactivation of the stringent response. Expression of these mutated *rel* genes resulted in a phenotype that was not distinguishable from the phenotype of the (p)ppGpp^0^ strain in terms of growth and gene expression pattern. Thus, the TGS and DC motifs are required for stringent response, while the ACT motif plays only a minor role.

### Co-immunoprecipitation (Co-IP) of Rel_*Sau*_

**Fig 7:**
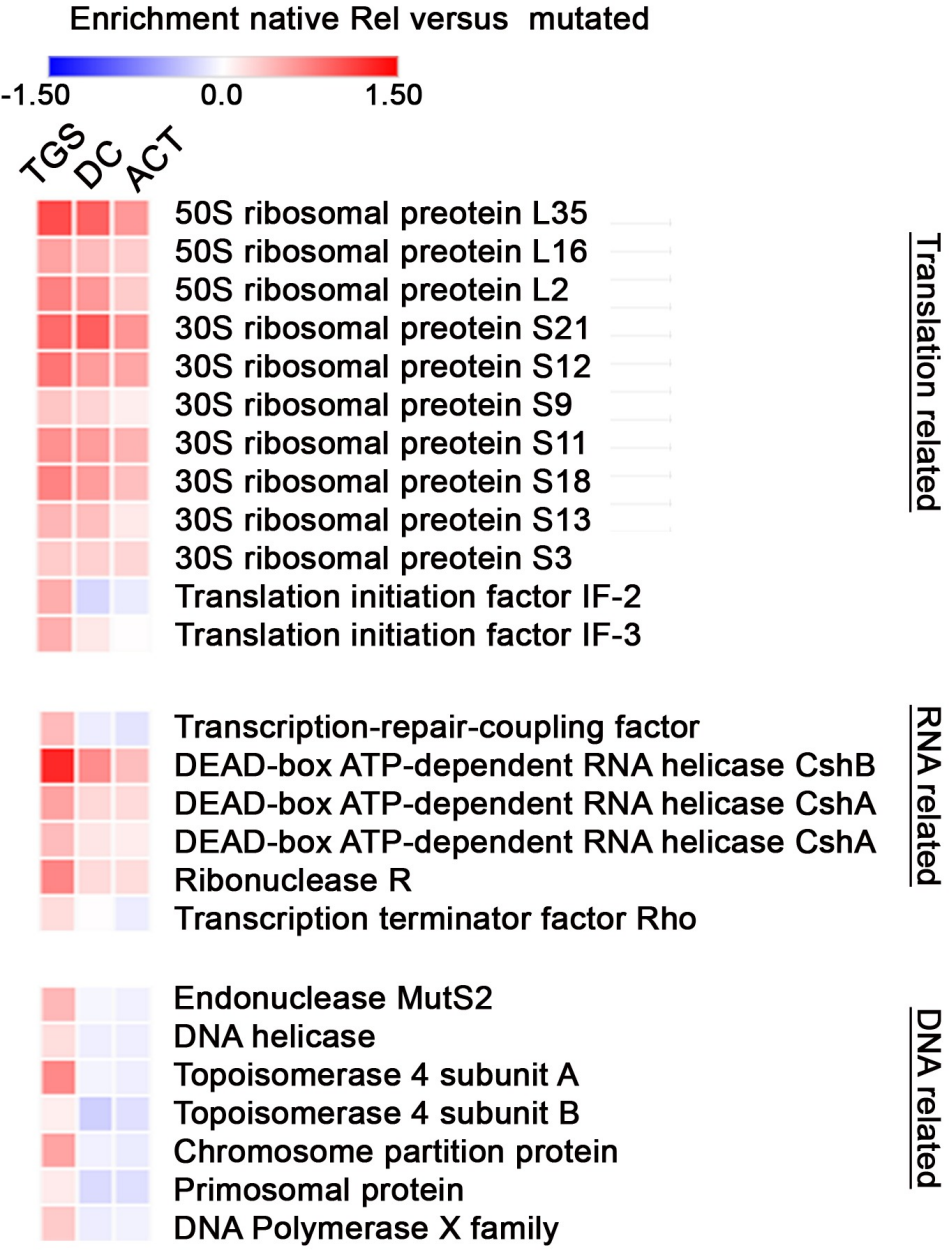
Influence of the CTD motifs on Rel_*Sau*_ interactions. *S*. *aureus* (p)ppGpp^0^ was complemented with native and mutated Rel_*Sau*_. Cell lysates were mixed with magnetic beads, coated with Rel_*Sau*_ antibodies and enriched proteins identified by MS. Proteins significantly (t-test difference > 0.05) less abundant using TGS mutated versus native Rel_*Sau*_ as bait are shown in the first column. The effects of the DC or ACT mutation on interaction with these proteins are shown in the second and third column, respectively.

We aimed to analyze the role of the conserved motifs within the CTD for interaction with cytosolic proteins. Therefore, we performed Co-IP experiments using whole-cell lysates of (p)ppGpp^0^ mutants expressing wild-type or mutated (ACT, DC and TGS see Fig 1A) versions of Rel_*Sau*_. For each pull-down experiment, the wild-type or mutant Rel_*Sau*_ was the most abundant protein detected, with no significant difference observed between wild type and mutant proteins (Data S1 Dataset) and the expression of all proteins was similar as shown by Western blot analysis (Blot in S1 Fig). Mainly ribosomal proteins were co-immuno-precipitated with native Rel_*Sau*_. When Rel_*Sau*_ with mutated TGS motif was used as bait significant less proteins were enriched (Fig 7 first column). Most of these putative TGS interacting proteins were also found to be effected when Rel_Sau_ harboring mutations in ACT or DC motifs were used, although to a lesser extent. Immuno-precipitated proteins that were strongly influenced by the TGS mutation are ribosomal proteins, proteins associated with RNA degradation and proteins involved in DNA-related pathways. In summary, the results indicate that all three motifs within the CTD work together to dock Rel_*Sau*_ onto the translational apparatus. The strongest interaction is mediated by TGS, whereas the ACT motif seems to have a low impact. The TGS motif seems to mediate also interaction with non-ribosomal proteins.

## Discussion

RSH enzymes are major players in the synthesis and hydrolysis of the second messenger (p)ppGpp. There is still limited information about the molecular switch that regulates the two activities, both present in long RSH enzymes. Here, we analyzed how the CTD of Rel_*Sau*_ influences the enzymatic activities *in vivo*. We showed that Rel_*Sau*_ exists primarily in a synthetase-OFF/hydrolase-ON conformation. Only under stringent growth conditions was the switching to the synthetase-ON conformation detectable, and this switching occurred only when the CTD possessed intact TGS and DC motifs.

In *S*. *aureus* and probably in other Firmicutes, Rel combines the functions of the two prototypic RSH enzymes, RelA and SpoT, from Proteobacteria. The synthetase activity is needed to elicit a stringent response phenotype, presumably via interaction with ribosomes and uncharged tRNA, as previously shown for RelA [24,25]. However, similar to SpoT, Rel_*Sau*_ also possesses strong hydrolase activity, which is necessary to counteract the (p)ppGpp production by the small synthetases RelP and RelQ present in Firmicutes. The equilibrium between these two activities needs to be tightly regulated in order to attain an appropriate level of (p)ppGpp based on the growth conditions. So far, potential differences between RelA, SpoT and Rel associated with the molecular switch could not be inferred from the sequence or *in vitro* analyses. *In vivo* activities of different RSH enzymes were mainly analyzed by heterologous expression in *E*. *coli*. These analyses indicated that without CTDs, RSH enzymes possess strong synthetase activity [21,44,45]. Similarly, Rel_*Sau*_, with or without the CTD, can complement an *E*. *coli relA/spoT* mutant, also indicating that (p)ppGpp synthesis can occur with or without the CTD. However, analysis of the same construct in the native background clearly showed that Rel_*Sau*_ lacking the CTD is tightly held in the hydrolase-ON state, and synthetase activity is detectable only in constructs that lack the hydrolase. Thus, Rel_*Sau*_, with or without the CTD, exhibits net (p)ppGpp synthetase activity when expressed in *E*. *coli* but net hydrolase activity when expressed in *S*. *aureus*. It would be interesting to see whether enzymes from other organisms show a similar discrepancy between *E*. *coli* and native backgrounds. Our results indicated that the enzymatic activity of Rel_*Sau*_ is influenced by species-specific interactions of the enzymatic NTD with unknown factors. To date, there is no evidence that the NTD alone interacts with the ribosome. Thus, other interaction partners or intracellular properties of the NTD should be elucidated in the future. An alternative possibility is that less (p)ppGpp is needed to complement the phenotype of a pppGpp mutant in *E*. *coli* allowing growth even if Rel_*Sau*_ has a weak synthetase. However, this is not supported by our in vitro results, showing that synthetase activity is only detectable when the hydrolase is mutated in constructs with or without CTD. Analysis of full-length or truncated Rel_*Sau*_ *in vitro* largely confirmed the results obtained with the in vivo data obtained in *S*. *aureus*. The presence of an intact hydrolase abrogates the synthetase activity. Synthesis of pppGpp was detectable only in hydrolase-deficient constructs. Moreover, we show that the CTD has an inhibitory effect on synthetase activity since truncated versions of Rel_*Sau*_ showed higher accumulation of the reaction products AMP and pppGpp compared to the full-length enzyme. However, the CTD had only a minor impact on the strong hydrolase activity of the purified enzymes. Interestingly, Rel_*Sau*_ preferentially hydrolyzes ppGpp over pppGpp. *In vivo*, it was shown that RelP and RelQ mainly produce ppGpp [35], which is toxic at high concentrations and requires efficient hydrolysis. This observation could explain the preference of Rel_*Sau*_ for ppGpp hydrolysis.

The Co-IP results indicate that Rel_*Sau*_ interacts with the translation machinery and that the TGS strongly influence this interaction. This is largely consistent with previous data obtained for RelA [24,25,28,29,30]. Among the 10 detected interacting ribosomal proteins, L16, S13, and S12 are homologous to *E*. *coli* proteins that have been previously identified to interact with RelA [28,29,30]. Of note, the TGS motif also seems to hamper the putative interaction of Rel_*Sau*_ with other proteins of the RNA and DNA pathways. Whether such interactions are specific and involved in the molecular function of Rel_*Sau*_ remains to be investigated.

The *in vivo* analyses combined with the Co-IP results provided some clues regarding the roles of the different motifs of the CTD of Rel_*Sau*_. Of the three motifs, the TGS motif showed the strongest effect, and the ACT motif showed the weakest effect, on (p)ppGpp activation and ribosomal interaction. Thus, the role of the ACT motif remains to be elucidated but seems to be minor. The TGS motif is clearly required for synthetase activation, most likely interacts with the ribosome to sense whether or not the tRNA in the A-site is aminoacetylated as shown for RelA [28,29,30,46]. Similar to the TGS motif, the DC motif was also found to be required for synthetase activity and to influence interactions with ribosomal proteins. DC has also been reported to interact with 23S rRNA ASF [28] which is critical to RelA activation in E. coli [46], presumably through stabilizing ribosome interaction.

This finding contradicts the simple model in which the DC motif causes oligomerization and thereby autoinhibition [31,32,33]. This would imply that DC mutation alleviates autoinhibition leading to increased synthase activity. In contrast, our data showed that the DC-mutated Rel_*Sau*_ is held in a synthetase-OFF state. Thus, our data support a model in which the DC motif participates in specific activation upon ribosomal contact, and that this interaction is involved in the intramolecular switch.

## Materials and methods

### Strains and growth conditions

Strains and plasmids are listed in the table in S1 Table. For strains carrying resistance genes, antibiotics (10 μg/ml erythromycin, 5 μg/ml tetracycline, 10 μg/ml chloramphenicol, and 100 μg/ml ampicillin) were used only in precultures. For the conditional mutant HG001-55, IPTG (final concentration of 0.5 mM) was added only in the preculture. *S*. *aureus* strains were grown in CYPG (10 g/l casamino acids, 10 g/l yeast extract, 5 g/l NaCl, 0.5% glucose and 0.06 M phosphoglycerate) medium [47]. Bacteria from an overnight culture were diluted to an initial optical density (OD_600_) of 0.05 in fresh medium and grown with shaking (220 rpm) at 37°C to the desired growth phase. Expression of cloned proteins was induced in exponential phase (OD_600_ = 0.3) with 0.1 μg/ml anhydrotetracycline (ATc) and in stringent conditions by addition of 0.3 μg/ml mupirocin. *E*. *coli* strains were grown in an overnight preculture in LB medium. Stringent conditions were applied by growing cells in modified M9 medium (33.7 mM NaHPO_4_, 22 mM KH_2_PO_4_, 8.55 mM NaCl, 9.35 mM NH_4_Cl, 1 mM MgSO_4_, 0.3 mM CaCl_2_, 1 μg/ml thiamine hydrochloride, 0.4% glycerol, 1 mM serine, 1 mM methionine and 1 mM glycine) [48]. For growth on solid media, single colonies grown on LB agar were streaked on M9 agar plates. For growth curve analyses, bacteria were inoculated to the desired OD_600_ (*S*. *aureus* initial OD_600_ = 0.05; *E*. *coli* initial OD_600_ = 0.1) in a 96-well plate, and growth was monitored in an Infinite M200 Pro microplate reader (Tecan).

### Construction of vectors for expression of full-length and truncated *rel*

All oligonucleotides are listed in S2 Table. ATc-inducible plasmids, derived from pCG248, were generated with a restriction enzyme cloning strategy. Amplicons and vector were digested with EcoRI restriction enzyme. Substitution of the hydrolase domain and the ACT, TGS and DC mutations were achieved by overlapping PCR. For expression of the *rel* constructs under the native promoter, the shuttle vector pCG443 was designed based on pJL77 [49]. pJL77 was digested with AscI and SphI to remove the previous insert, including the promoter. The *rel* promoter was amplified from genomic DNA, digested using the same restriction enzymes, and ligated to generate pCG443. Full-length, truncated and mutated versions of *rel* were subcloned from the pCG248 plasmids into AscI-digested pCG443 by Gibson assembly [50].

All inserts were verified by sequencing (4base lab AG advanced molecular analysis), electroporated into the restriction-deficient *S*. *aureus* strain RN4220, and then transduced into the final *S*. *aureus* strains. All *S*. *aureus* strains were tested by PCR for the presence of the correct plasmid.

For expression in *E*. *coli*, different derivatives of *rel* were cloned into the EcoRI site of pBAD30 via Gibson assembly using the oligonucleotides listed in Table S2. Resulting vectors were verified by sequencing and moved to MG1655 *E*. *coli* strains (wild type and *relA/spoT* mutant) [51,52].

For protein purification, different *rel* derivatives were subcloned from the pCG248-based plasmids into BamHI-digested pET15b using Gibson assembly.

### Generation of *S*. *aureus* mutant strains

The markerless *rel* CTD-deletion mutant was obtained using the ATc-inducible suicide vector pBASE6 [34]. Deletion was introduced by overlapping PCR with the primers listed in S2 Table, and the amplicon was cloned into BglII- and SalI-digested pBASE6 by Gibson assembly. The resulting plasmid was verified by sequencing and electroporated into RN4220, from which the plasmid was transduced into HG001. Mutagenesis was performed as described previously [34]. Mutation was verified by PCR.

### RNA isolation and Northern blot analysis

RNA isolation and Northern blot analysis were performed as described previously [53]. Briefly, 5 ml of bacteria were collected at the desired time point (30 minutes after induction) and centrifuged. The pellet was resuspended in 1 ml of TRIzol reagent (Thermo Fisher Scientific) with 0.5 ml of zirconia/silica beads (0.1-mm diameter) and lysed using a high-speed homogenizer (Thermo Fisher Scientific). RNA was isolated following the instructions provided by the TRIzol manufacturer. For the detection of specific transcripts on the Northern blot, digoxigenin-labeled probes were generated using the DIG-labeling PCR Kit as described by the manufacturer (Roche Life Science).

### Purification of different Rel_*Sau*_ constructs

*E*. *coli* BL21 (DE3) (New England Biolabs) cells that were freshly transformed with plasmids carrying full-length *rel* constructs were grown for 16 hours at room temperature under constant shaking (150 rpm) in LB medium supplemented with D(+)-lactose-monohydrate (12.5 g/l) and ampicillin (100 μg/ml). Cells were harvested (20 minutes, 3000 x g, 4°C) and resuspended in ice-cold high-KCl buffer A (20 mM HEPES (pH 7.4), 20 mM NaCl, 20 mM MgCl_2_, 1 M KCl, 30% (v/v) glycerol, and 40 mM imidazole) supplemented with 10 μg/ml DNAse and cOmplete protease inhibitor cocktail (Roche). Cells were lysed by a French press at 1000 psi. The lysate was centrifuged (50,000 x g, 45 minutes, 4°C), and the clear supernatant was filtered (0.22-μm pore size) before being loaded onto a 1-ml HisTrap HP column (GE Healthcare Life Sciences) equilibrated with high-KCl buffer A. Purification was performed with an ÄKTA purification system (GE Healthcare Life Sciences), and elution was carried out with an imidazole gradient to a final concentration of 500 mM. Fractions were analyzed by SDS-PAGE, and the fractions containing the protein of interest were collected and concentrated to 5 ml with an Amicon Ultracel-50K ultracentrifugal device, with a cut-off of 50 kDa (Merck Millipore). Protein was further purified by size-exclusion chromatography (HiLoad 16/600 Superdex 200 pg, GE Healthcare Life Sciences). The size-exclusion column was previously equilibrated with ice-cold high-KCl SEC buffer (20 mM HEPES (pH 7.0), 20 mM NaCl, 20 mM MgCl_2_, 1 M KCl, and 30% (v/v) glycerol). Protein-containing fractions were pooled, concentrated by ultra-filtration with a 50-kDa cut-off, aliquoted and stored at -80°C. For purification of CTD-truncated constructs, the same procedure was followed using different buffers: low-KCl buffer A (20 mM HEPES (pH 7.4), 200 mM NaCl, 20 mM MgCl_2_, 20 mM KCl, 30% (v/v) glycerol, and 40 mM imidazole) for affinity purification and low-KCl SEC buffer (20 mM HEPES (pH 7.0), 200 mM NaCl, 20 mM MgCl_2_, 20 mM KCl, and 30% (v/v) glycerol) for size exclusion. For concentration of truncated Rel_*Sau*_, Amicon Ultracel-30K (Merck Millipore) was used.

### *In vitro* assay for enzymatic activity and HPLC-MS analysis

Synthetase assays were performed in reaction buffer (20 mM HEPES (pH 7.0), 200 mM NaCl, 20 mM MgCl_2_, and 20 mM KCl) with 1 mM ATP, 1 mM GTP and 2 μM purified enzyme. Hydrolase assays were performed in the same reaction buffer with 1 mM ppGpp or 1 mM pppGpp (both from Jena Biosciences) and 0.1 μM purified enzyme. Assays were performed at 37°C; aliquots were taken at the indicated times; and the enzyme reactions were stopped by addition of an equal volume of chloroform. The mixtures were briefly vortexed and centrifuged (3 minutes, 11,000 × g). The aqueous phase containing the nucleotides was collected and stored at -20°C prior to analysis. Nucleotide analysis was performed using an ESI-TOF mass spectrometer (micrO-TOF II, Bruker) operated in negative-ion mode and connected to an UltiMate 3000 high-performance liquid chromatography (HPLC) system (Dionex). 5 μl of each sample at 10°C was injected onto the SeQuant ZIC-pHILIC column (Merck, PEEK 150 × 2.1 mm, 5 μm), and the system was run at 30°C as previously described [43]. The following 40-minute gradient program was used at a flow rate of 0.2 ml/min: 5 minutes of 82% buffer A (CH_3_CN) and 18% buffer B (100 mM (NH_4_)_2_CO_3_, pH 9.2); 25 minutes of a linear gradient to 42% buffer A; and finally, 10 minutes of 82% buffer A. The DataAnalysis program (Bruker) was used to present the nucleotide masses as extracted-ion chromatograms, and the peak areas were calculated and quantified with Prism 5 (GraphPad). Dilution series of commercially available nucleotides ppGpp (m/z, 601.95), pppGpp (m/z, 681.92) and AMP (m/z, 346.06) were used for calibration to quantify the amounts of nucleotides in the reactions.

### Co-IP of Rel_*Sau*_ with native *S*. *aureus* proteins

To generate Rel_*Sau*_-specific antibodies, 0.5 mg of purified full-length protein was sent to Davids Biotechnologie GmbH to generate antiserum and affinity-purified IgG. The specificity of the IgG was verified by Western blot analysis (Blot in S1 Fig). For Co-IP, bacteria were grown in 100 ml of CYPG medium to an OD_600_ of 1 and centrifuged (5,000 μ g, 5 minutes). The pellet was washed 2 times with PBS and resuspended in 500 μl of cold Co-IP buffer (20 mM HEPES (pH 7.0), 200 mM NaCl, 20 mM MgCl, 20 mM KCl, 0.5 mM DTT, 0.2% (v/v) Tween 20 and cOmplete protease inhibitor cocktail). The resuspended pellet was lysed with 0.5 ml of zirconia-silica beads (0.1 mm diameter) using a high-speed homogenizer (two times, 6,500 rpm, 20 s). Lysed cells were centrifuged for 1 hour at 14,000 x g at 4°C, and the supernatant was aliquoted (100 μl) and frozen at -80°C. Co-IP was performed with Dynabeads (Thermo Fisher Scientific) following the manufacturer’s instructions with some minor modifications. Briefly, 50 μl of Dynabeads slurry was used for each sample. The storage solution was removed, and the beads were incubated with 30 μg of Anti-Rel_*Sau*_ IgG resuspended in PBS (pH 7.4) with 0.02% Tween 20 for 30 minutes at room temperature under constant rotation. Coated beads were pelleted using a magnetic rack; the supernatant was removed; and 100 μl of the cell lysates were added and incubated for 30 minutes at room temperature under constant rotation. After incubation, the beads were gently washed 3 times with Co-IP buffer using a magnetic rack. Washing solution was removed, and the beads were resuspended in SDS sample buffer, boiled at 95°C for 5 minutes, and run approximately 1 cm into an SDS-PAGE gel. The gel slice was subsequently analyzed by mass spectrometry.

### Quantitative label-free proteomics

Three biological replicates of (p)ppGpp^0^ complemented with WT and mutant Rel_*Sau*_ were analyzed. Gel slices were digested as described previously [54]. Peptide mixtures were then separated on an EasyLC nano-HPLC (Proxeon Biosystems) coupled to an LTQ Orbitrap Elite mass spectrometer (Thermo Fisher Scientific) as described elsewhere [55] with the following modifications: peptides were eluted with an 87-min segmented gradient of 5–33–90% HPLC solvent B (80% acetonitrile in 0.5% acetic acid). Each sample was run in triplicate. The acquired MS spectra were processed with the MaxQuant software package, version 1.5.2.8 [56] with the integrated Andromeda search engine [57] as described previously [55]. Database searches were performed against a target-decoy *S*. *aureus* all-strains database obtained from UniProt, containing 126,225 protein entries and 248 commonly observed contaminants. The label-free algorithm was enabled, as was the “match between runs” option [58]. Label-free quantification (LFQ) protein intensities from the MaxQuant data output were used for relative protein quantification. Downstream bioinformatic analysis (ANOVA and two-sample t-tests) was performed using the Perseus software package, version 1.5.0.15. P < 0.05 was considered to be statistically significant. For the heatmap, among the 4 different proteins, those that showed significant differences according to ANOVA were selected (Data in S1 Dataset). For these selected candidates, the t-test differences, indicating changes in the amount, were calculated between the protein immunoprecipitated with WT or mutant Rel_*Sau*_ and plotted on the heatmap.

### Statistical analysis

The results for the growth and *in vitro* analyses represent the mean ± SD of at least three biological replicates. Significance was calculated using Prism 5 by one-way ANOVA with Bonferroni correction.

## Supporting information

S1 Fig Immunoblot showing the expression level of the different Rel_*Sau*_ constructs. *S*. *aureus* (p)ppGpp^0^ complemented with Rel_*Sau*_, wild type and different domain mutants, under the native promoter. Strains were grown in rich medium to OD_600_ = 1 and then harvested. For Worthern analysis, lysate was obtained and the different Rel_*Sau*_ constructs were detected with anti-Rel specific antibody.
(TIF)

S1 Table Strains and plasmids. (DOCX)

S2 Table Oligonucleotides. (DOCX)

S1 Dataset Comparison of Co-immune-precipitation results using Rel_*Sau*_ and TGS, ACT and DC mutated Rel_*Sau*_ as bait. (XLSX)

S1 Protocol Protocol for Rel-specific Western blot. (DOCX)
