# Supplementary figures and images for: Regulation of the opposing (p)ppGpp synthetase and hydrolase activities in a bifunctional RelA/SpoT homologue from Staphylococcus aureus
Source: PLoS Genet. 2018 Jul 9;14(7):e1007514. doi: 10.1371/journal.pgen.1007514 (PMC6053245; doi:10.1371/journal.pgen.1007514)

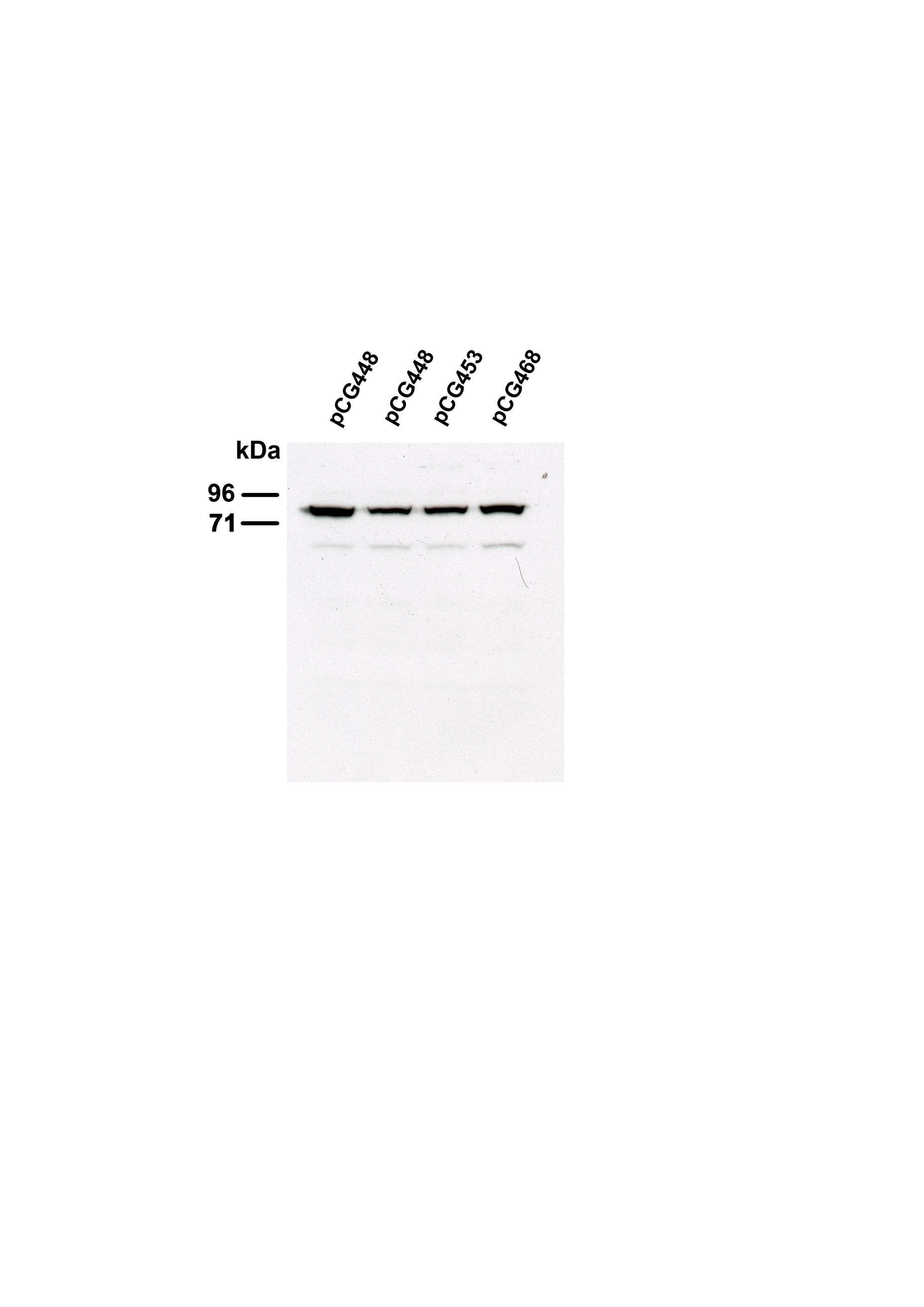

Supplement: S1 Fig — S. aureus (p)ppGpp0 complemented with RelSau, wild type and different domain mutants, under the native promoter. Strains were grown in rich medium to OD600 = 1 and then harvested. For Worthern analysis, lysate was obtained and the different RelSau constructs were detected with anti-Rel specific antibody. (TIF) [file pgen.1007514.s001.tif]
