# Supplementary material for: Regulation of the opposing (p)ppGpp synthetase and hydrolase activities in a bifunctional RelA/SpoT homologue from Staphylococcus aureus
Source: PLoS Genet. 2018 Jul 9;14(7):e1007514. doi: 10.1371/journal.pgen.1007514 (PMC6053245; doi:10.1371/journal.pgen.1007514)
Supplement: S1 Table — (DOCX) [file pgen.1007514.s002.docx]

**Table S1: Strains and Plasmids**

| **Strains** | **Description** | **Source / Reference** |
| --- | --- | --- |
| ***E. coli*** |  |  |
| Top10 | mcrA, Δ(mrr-hsdRMS-mcrBC), Phi80lacZ(del)M15, ΔlacX74, deoR, recA1, araD139, Δ(ara-leu)7697, galU, galK, rpsL(SmR), endA1, nupG, competent for plasmid trasformation | INVITROGEN |
| MG1655 | F^-^, lambda^-^, rph-1 | (Blattner et al., 1997) |
| MG1655 *relA/spoT* | F^-^, lambda^-^, rph-1 relA/spoT deletion | (Xiao et al., 1991) |
| BL21 (DE3) | fhuA2 [lon] ompT gal (λ DE3) [dcm] ∆hsdS λ DE3 = λ sBamHIo ∆EcoRI-B int::(lacI::PlacUV5::T7 gene1) i21 ∆nin5, competent for protein expression | NEB |
| **S. aureus** |  |  |
| RN4220 | Restriction-deficient derivate of 8325-4, rK^−^mK^+^ | (Kreiswirth et al., 1983) |
| HG001 | RN1 derivative, rsbU repaired, tcaR | (Pohl et al., 2009; Herbert et al., 2010) |
| HG001-531 | HG001 with Rel C-terminus deletion | this work |
| HG001-55 | HG001 with *rel* gene under Pspac promoter | (Geiger et al., 2010) |
| HG001-229-230-263 | HG001 *relP/relQ/rel* deletion, named as (p)ppGpp^0^ | (Geiger et al., 2014) |
| HG001-86 | HG001 *rel_syn_* mutation | (Geiger et al., 2010) |

| **Plasmids** | **Description** | **Source / Reference** |
| --- | --- | --- |
| pET15b | protein expression vector, Ampicillin | Novagen |
| pCG511 | Rel native in protein expression vector pET15b | this work |
| pCG512 | Rel N-terminus in protein expression vector pET15b | this work |
| pCG550 | Rel hydrolase mutated in protein expression vector pET15b | this work |
| pCG551 | Rel hydrolase mutated N-terminus in protein expression vector pET15b | this work |
| pCG248 | anhydrotetracyclin inducible vector, Chloramphenicol | (Helle et al., 2011; Schroder et al., 2013) |
| pCG390 | Rel native in pCG248 | this work |
| pCG326 | Rel N-terminus in pCG248 | this work |
| pCG328 | Rel hydrolase in pCG248 | (Geiger et al., 2014) |
| pCG327 | Rel hydrolase mutated N-terminus in pCG248 | this work |
| pCG259 | relQ in pCG248 | (Geiger et al., 2014) |
| pCG443 | Rel native protmoter vector, Erythromycin | this work |
| pCG448 | Rel native in pCG443 | this work |
| pCG449 | Rel N-terminus in pCG443 | this work |
| pCG452 | Rel act domain mutated in pCG443 | this work |
| pCG453 | Rel D625R C626F substitution in pCG443 | this work |
| pCG468 | Rel tgs domain mutated in pCG443 | this work |
| pBAD30 | Arabinose inducible vector, Ampicillin | Guzman et al. 1995 |
| pCG489 | Rel native in pBAD30 | this work |
| pCG490 | Rel N-terminus in pBAD30 | this work |
| pCG527 | Rel synthetase mutated in pBAD30 | this work |
| pCG528 | Rel synthtase mutated N-terminus inpBAD30 | this work |
| pCG624 | Rel hydrolase mutated in pBAD30 | this work |
| pCG625 | Rel hydrolase mutated N-terminus in pBAD30 | this work |
| pBASE | Anhydrotetracyclin inducible suicide mutagenesis vector, derivative from pKOR1 | (Geiger et al., 2012) |
| pCG531 | Rel C-terminus deletion in pBASE | this work |
| pJL77 | Venus fluorence protein under agr promoter | (Liese et al., 2013) |
| pCG436 | Rel act domain mutated in pCG248 | this work |
| pCG441 | Rel D625R C626F substitution in pCG248 | this work |
| pCG442 | Rel tgs domain mutated in pCG248 | this work |

Blattner, F.R., Plunkett, G., 3rd, Bloch, C.A., Perna, N.T., Burland, V., Riley, M. et al. (1997) The complete genome sequence of Escherichia coli K-12. *Science* **277**: 1453-1462.

Geiger, T., Kastle, B., Gratani, F.L., Goerke, C., and Wolz, C. (2014) Two small (p)ppGpp synthases in Staphylococcus aureus mediate tolerance against cell envelope stress conditions. *J Bacteriol* **196**: 894-902.

Geiger, T., Goerke, C., Fritz, M., Schafer, T., Ohlsen, K., Liebeke, M. et al. (2010) Role of the (p)ppGpp synthase RSH, a RelA/SpoT homolog, in stringent response and virulence of *Staphylococcus aureus*. *Infect Immun* **78**: 1873-1883.

Geiger, T., Francois, P., Liebeke, M., Fraunholz, M., Goerke, C., Krismer, B. et al. (2012) The stringent response of *Staphylococcus aureus* and its impact on survival after phagocytosis through the induction of intracellular PSMs expression. *PLoS Pathog* **8**: e1003016.

Helle, L., Kull, M., Mayer, S., Marincola, G., Zelder, M.E., Goerke, C. et al. (2011) Vectors for improved Tet repressor dependent gradual gene induction or silencing in *Staphylococcus aureus*. *Microbiology*.

Herbert, S., Ziebandt, A.K., Ohlsen, K., Schafer, T., Hecker, M., Albrecht, D. et al. (2010) Repair of global regulators in *Staphylococcus aureus* 8325 and comparative analysis with other clinical isolates. *Infect Immun* **78**: 2877-2889.

Kreiswirth, B.N., Lofdahl, S., Betley, M.J., O'Reilly, M., Schlievert, P.M., Bergdoll, M.S., and Novick, R.P. (1983) The toxic shock syndrome exotoxin structural gene is not detectably transmitted by a prophage. *Nature* **305**: 709-712.

Liese, J., Rooijakkers, S.H., van Strijp, J.A., Novick, R.P., and Dustin, M.L. (2013) Intravital two-photon microscopy of host-pathogen interactions in a mouse model of *Staphylococcus aureus* skin abscess formation. *Cell Microbiol* **15**: 891-909.

Pohl, K., Francois, P., Stenz, L., Schlink, F., Geiger, T., Herbert, S. et al. (2009) CodY in *Staphylococcus aureus*: a regulatory link between metabolism and virulence gene expression. *J Bacteriol* **191**: 2953-2963.

Schroder, W., Goerke, C., and Wolz, C. (2013) Opposing effects of aminocoumarins and fluoroquinolones on the SOS response and adaptability in Staphylococcus aureus. *J Antimicrob Chemother* **68**: 529-538.

Xiao, H., Kalman, M., Ikehara, K., Zemel, S., Glaser, G., and Cashel, M. (1991) Residual guanosine 3',5'-bispyrophosphate synthetic activity of *rel*A null mutants can be eliminated by *spo*T null mutations. *J Biol Chem* **266**: 5980-5990.
