# Supplementary material for: Regulation of the opposing (p)ppGpp synthetase and hydrolase activities in a bifunctional RelA/SpoT homologue from Staphylococcus aureus
Source: PLoS Genet. 2018 Jul 9;14(7):e1007514. doi: 10.1371/journal.pgen.1007514 (PMC6053245; doi:10.1371/journal.pgen.1007514)
Supplement: S2 Table — (DOCX) [file pgen.1007514.s003.docx]

**Table S2: Oligonucleotides**

| **Final Vector** | **Name** | **Sequence** | **Template** |
| --- | --- | --- | --- |
| pCG531 | relLC1-for relCtermdel-rev relCtermdel-for relseq4-rev relCterm.Gbs.pBASE-for relCterm.Gbs.pBASE-rev | GGCAATCGCAATAAATAAC CTTGTTACTGTATAAACATCTTTGTCACTCTGTAAGTCATAT  GATGTTTATACAGTAACAAG  CATTTCAAGAATACTAACGG  tacccgggctagcgcgcaTATTGATAACGGCTCTTCGT  catgcaagcttgatatcgCACCTTTCTCAACTTTATATATGACTGGA | HG001 |
| pCG511 | RSH.pET15b.3-for RSH.Gbs.pET-15b-rev | ggcagccatatgctcgagATGAACAACGAATATCCATATAGTGCA  gggctttgttagcagccgCTAGTTCCAAACTCTTGTTACTGT | HG001 |
| pCG512 | RSH.pET15b.3-for  RSHN-term.pET15b.3-rev | ggcagccatatgctcgagATGAACAACGAATATCCATATAGTGCA  ttcgggctttgttagcagccgctaACTCTGTAAGTCATATTTTAAGGTTTCCA | HG001 |
| pCG550 | RSH.pET15b.3-for RSH.Gbs.pET-15b-rev | ggcagccatatgctcgagATGAACAACGAATATCCATATAGTGCA  gggctttgttagcagccgCTAGTTCCAAACTCTTGTTACTGT | pCG328 |
| pCG551 | RSH.pET15b.3-for  RSHN-term.pET15b.3-rev | ggcagccatatgctcgagATGAACAACGAATATCCATATAGTGCA  ttcgggctttgttagcagccgctaACTCTGTAAGTCATATTTTAAGGTTTCCA | pCG328 |
| pCG390 | EcoRIrel1151for EcoRIrel-rev | aaaagaattcGTACCTAAATCATTGTTTAAGGCG  ccccgaattcCCGATACCGACTAATAAACAATA | HG001 |
| pCG326 | EcoRIrel1151for EcoRI-N-term+STOP | aaaagaattcGTACCTAAATCATTGTTTAAGGCG  ccccgaattcTTACTCTGTAAGTCATATTTTAA | HG001 |
| pCG327 | EcoRIrel1151for EcoRI-N-term+STOP | aaaagaattcGTACCTAAATCATTGTTTAAGGCG  ccccgaattcTTACTCTGTAAGTCATATTTTAA | pCG328 |
| pCG443 | SphI.RSH.Promoter-for AscI.Rel.Promoter-rev | gcccgcatgcTAAATAATATAATTTTATCA  aattggcgcgccATTTATTACTTCGCCTTAAA | HG001 |
| pCG448 | RSH.Gbs.443-for RSH.Gbs.443-rev | gtttaaggcgaagtaataaatggATGAATGGGGTGTATCATAT  gtatttattatgcattagaataggCTAGTTCCAAACTCTTGTTACTGT | pCG390 |
| pCG449 | RSH.Gbs.443-for RSHN-term.Gbs.443-rev | gtttaaggcgaagtaataaatggATGAATGGGGTGTATCATAT  gtatttattatgcattagaataggctaCTCTGTAAGTCATATTTTAAGGTTTCCA | pCG390 |
| pCG452 | RSH.Gbs.443-for RSH.Gbs.443-rev | gtttaaggcgaagtaataaatggATGAATGGGGTGTATCATAT  gtatttattatgcattagaataggCTAGTTCCAAACTCTTGTTACTGT | pCG436 |
| pCG453 | RSH.Gbs.443-for RSH.Gbs.443-rev | gtttaaggcgaagtaataaatggATGAATGGGGTGTATCATAT  gtatttattatgcattagaataggCTAGTTCCAAACTCTTGTTACTGT | pCG441 |
| pCG468 | RSH.Gbs.248 - for RSH.Gbs.248 - rev | gtttaaggcgaagtaataaatggATGAATGGGGTGTATCATAT  gtatttattatgcattagaataggCTAGTTCCAAACTCTTGTTACTGT | pCG442 |
| pCG436 | pCNseq-for3 ACTmutrel-for ACT-mutrel-rev pCNseq-rev  RSH.Gbs.248-for RSH.Gbs.248-rev | TGACACTCTATCATTGATAG  GAATTGCAGTCAAATAATAATGAAGTACTACAAGCTGTT  ATTATTTGACTGCAATTCATACGCAGTTACCTCTAAATC  TGACACTCTATCATTGATAG ttaacagatctgagctcgatgaatggggtgtatcatat  ttagaataggcgcgcctgctagttccaaactcttgttactgt | HG001 |
| pCG441 | pCNseq-for3 mutrelDC625RF1-rev mutrelDC625RF-for pCNseq-rev  RSH.Gbs.248-for RSH.Gbs.248-rev | TGACACTCTATCATTGATAG  TGACACTCTATCATTGATAG  AAAGTACATCGCACTAGGTTTCCAAATATTAAGAAC  GTTCTTAATATTTGGAAACCTAGTGCGATGTACTTT  ttaacagatctgagctcgATGAATGGGGTGTATCATAT  ttagaataggcgcgcctgCTAGTTCCAAACTCTTGTTACTGT | HG001 |
| pCG442 | relA906-for  TGSmutrel1-rev newTGSmutrel-for Relseq4-rev RSH.Gbs.248 - for RSH.Gbs.248 - rev | gatgattgagcattatacct  CTGCACTTCTATGCGGACTTTTTGGAAACCAGGTAATAAG  ATGATTGGTGCC CTGCACTTCTATGCGGACTTTTTGGAAACCGGTAATAAGATGATTGGTGCC ccgttagtattcttgaaatg  ttaacagatctgagctcgATGAATGGGGTGTATCATAT  ttagaataggcgcgcctgCTAGTTCCAAACTCTTGTTACTGT | HG001 |
| pCG489 | RSH1ATG.pBAD30for RSH.pBAD30-rev | ctctactgtttctccatacccgtttttttgggctagcgaaaataaggaggaaaaaaaaATGAATGGGGTGTATCATAT ccccgggtaccgagctcgCTAGTTCCAAACTCTTGTTACTGT | HG001 |
| pCG490 | RSH1ATG.pBAD30for RSH.Nterm.pBAD30-rev | ctctactgtttctccatacccgtttttttgggctagcgaaaataaggaggaaaaaaaaATGAATGGGGTGTATCATAT ggatccccgggtaccgagctcgctagCTCTGTAAGTCATATTTTAAGGTTTCCA | HG001 |
| pCG527 | RSH1ATG.pBAD30for RSH.pBAD30-rev | ctctactgtttctccatacccgtttttttgggctagcgaaaataaggaggaaaaaaaaATGAATGGGGTGTATCATAT ccccgggtaccgagctcgCTAGTTCCAAACTCTTGTTACTGT | HG001-86 |
| pCG528 | RSH1ATG.pBAD30for RSH.pBAD30-rev | ctctactgtttctccatacccgtttttttgggctagcgaaaataaggaggaaaaaaaaATGAATGGGGTGTATCATAT ggatccccgggtaccgagctcgctagCTCTGTAAGTCATATTTTAAGGTTTCCA | HG001-86 |
| pCG624 | RSH1ATG.pBAD30for RSH.pBAD30-rev | ctctactgtttctccatacccgtttttttgggctagcgaaaataaggaggaaaaaaaaATGAATGGGGTGTATCATAT ccccgggtaccgagctcgCTAGTTCCAAACTCTTGTTACTGT | pCG328 |
| pCG625 | RSH1ATG.pBAD30for RSH.pBAD30-rev | ctctactgtttctccatacccgtttttttgggctagcgaaaataaggaggaaaaaaaaATGAATGGGGTGTATCATAT ggatccccgggtaccgagctcgctagCTCTGTAAGTCATATTTTAAGGTTTCCA | pCG328 |
