# Supplementary material for: Regulation of the opposing (p)ppGpp synthetase and hydrolase activities in a bifunctional RelA/SpoT homologue from Staphylococcus aureus
Source: PLoS Genet. 2018 Jul 9;14(7):e1007514. doi: 10.1371/journal.pgen.1007514 (PMC6053245; doi:10.1371/journal.pgen.1007514)
Supplement: S1 Protocol — (DOCX) [file pgen.1007514.s005.docx]

**S1 Protocol. Western blot analysis.** 10 ml OD_600_:1 of bacteria was collected and centrifuged. The pellet was washed in 5 ml of TE buffer and then re-suspended in 500 µl Lysis Buffer (TE-buffer + cOmplete protease inhibitor cocktail (Roche)) with 0.5 ml of zirconia/silica beads (0.1-mm diameter) and lysed using a high-speed homogenizer (Thermo Fisher Scientific). Samples were re-suspended 1:1 in Sample Buffer (500 µl of Runblue LDS (Expedeon), 500 µl H_2_0 and 0,008 g DTT) and incubated for 5 minutes at 95 °C. For each sample, 20 µl were loaded onto a SDS 12% PAGE gel (Expedeon) and protein were separated at 90 V over 1 hour and 20 minutes. Gel was blotted onto PVDF-membrane (immune-blot, Bio Rad), at 0,35 A for 1h. Membrane was blocked with Blocking buffer (10 ml TBST, 5% milk) for 1 h at room temperature. Samples detection was performed with anti-Rel*_Sau_* IgG (1:5000 dilution in 10 ml TBST, 1% milk) for 1 h at room temperature and Horseradish Peroxidase-Conjugated goat anti-rabbit IgG (1:10000 dilution in 10 ml TBST, 1% milk) (Southern Biotech Birmingham).
